# The simulation model of growth and cell divisions for the root apex with an apical cell in application to *Azolla pinnata*

**DOI:** 10.1007/s00425-013-1950-9

**Published:** 2013-08-30

**Authors:** Anna Piekarska-Stachowiak, Jerzy Nakielski

**Affiliations:** Department of Biophysics and Morphogenesis of Plants, University of Silesia, Jagiellońska 28, 40-032 Katowice, Poland

**Keywords:** Apical cell, *Azolla*, Cell divisions, Principal directions, Root growth, Simulation model, Tensor approach

## Abstract

In contrast to seed plants, the roots of most ferns have a single apical cell which is the ultimate source of all cells in the root. The apical cell has a tetrahedral shape and divides asymmetrically. The root cap derives from the distal division face, while merophytes derived from three proximal division faces contribute to the root proper. The merophytes are produced sequentially forming three sectors along a helix around the root axis. During development, they divide and differentiate in a predictable pattern. Such growth causes cell pattern of the root apex to be remarkably regular and self-perpetuating. The nature of this regularity remains unknown. This paper shows the 2D simulation model for growth of the root apex with the apical cell in application to *Azolla pinnata*. The field of growth rates of the organ, prescribed by the model, is of a tensor type (symplastic growth) and cells divide taking principal growth directions into account. The simulations show how the cell pattern in a longitudinal section of the apex develops in time. The virtual root apex grows realistically and its cell pattern is similar to that observed in anatomical sections. The simulations indicate that the cell pattern regularity results from cell divisions which are oriented with respect to principal growth directions. Such divisions are essential for maintenance of peri-anticlinal arrangement of cell walls and coordinated growth of merophytes during the development. The highly specific division program that takes place in merophytes prior to differentiation seems to be regulated at the cellular level.

**Electronic supplementary material:**

The online version of this article (doi:10.1007/s00425-013-1950-9) contains supplementary material, which is available to authorized users.

## Introduction

**Fig. 1:**
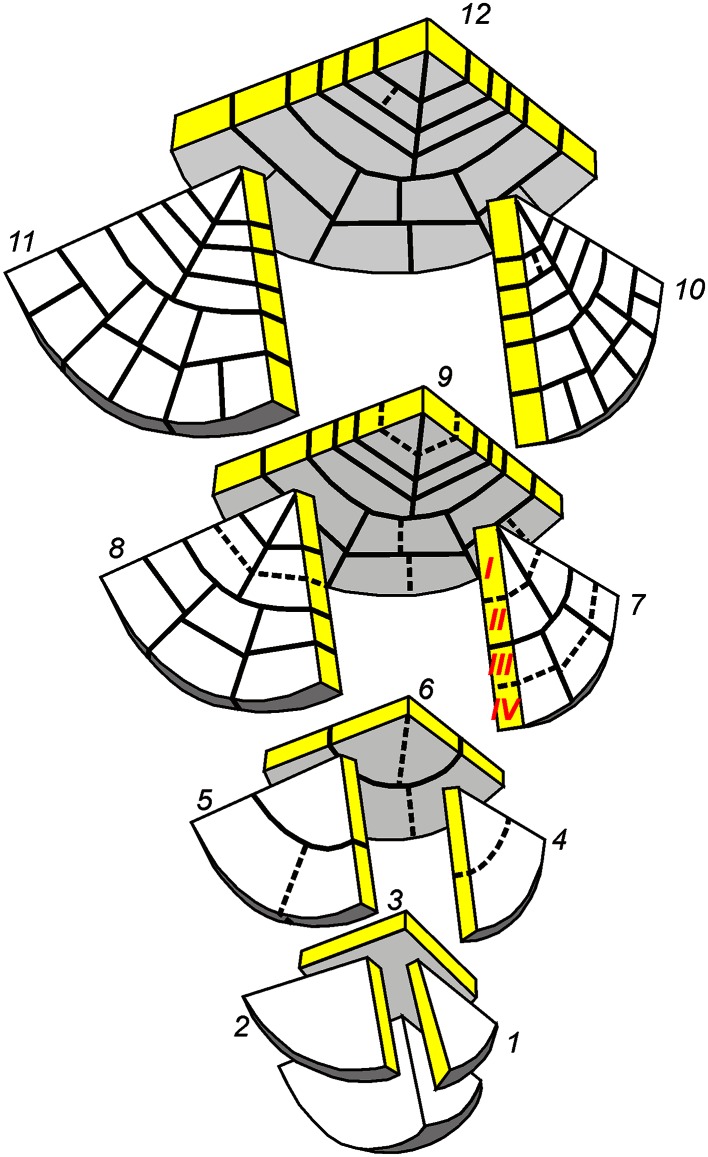
Schematic representation of formative divisions of *A. pinnata* root apex during twelve cell cycles durations (after Gunning 1982). In successive merophytes from 1 to 12 the longitudinal-tangential and longitudinal-radial divisions are shown; *dashed lines* represent new division in the sequence. The cell rows numbered in the merophyte 7 are considered in the text

Roots and shoots of pteridophytes comprise apical cells which are “stem cells” in which they renew themselves and produce a derivative every time they divide (Gunning 1982; Barlow 1997). Each derivative becomes added to the apex as a new construction unit called merophyte, within which set sequences of cell division, expansion and differentiation occur. In roots of *Azolla* (Gunning et al. 1978) and *Equisetum* (Gifford and Kurth 1982), a single apical cell (AC) located at the pole of the root proper occurs. The apical cell has a tetrahedral shape; its three faces are oriented proximally, while the fourth one which is a part of the root/cap border is oriented distally. During ontogenesis, the AC divides always asymmetrically. The derivative cut off from the AC distal face contributes to the root cap, while the derivatives from the three proximal division planes give rise to the proper root. Divisions along proximal faces are sequential and successive merophytes overlap with one another forming a strict merophyte helix arranged into 120° sectors along the root axis. In each new merophyte, formative divisions of the early longitudinal-tangential and longitudinal-radial types are observed in a definite sequence (Fig. 1). These formative divisions lead to determination of precursors of particular tissues which are defined starting from the outer tissues in the following order: epidermis and outer cortex, inner cortex and endodermis, pericycle and inner stele. Once the files are established, transverse divisions in the files take place and next differentiation occurs. As the AC divisions are predictable and the proliferation of cells in merophytes is highly specific (each cell type has its own program of divisions prior to terminal differentiation), the root apex has a very precise construction and its cell pattern is remarkably regular (Gunning et al. 1978). The nature of such regularity remains unknown.

The apical cell, though usually identified by morphological criteria, plays an important functional role (Bierhorst 1977). On the one hand, it is an ultimate source of all cells of the root apex and can be regarded as a single-cell meristem (Korn 1993). On the other hand, it works as a regulatory site and formative center in histogenesis and organogenesis. Positional signaling seems to play a role for such function (Gunning et al. 1978; Barlow 1991; Hou and Blancaflor 2009). However, how the AC specific division sequence is determined and maintained throughout the root ontogeny is still an open question.

The root apices, like other plant organs, grow symplastically (Priestley 1930; Erickson 1986). During such growth, considered typical for plants, cells are displaced in a coordinated way, preserving their mutual contacts. A description of the symplastic growth is based on the continuity condition (Gandar 1980; Silk 1984). It is assumed that at the organ level, there exists a field of the displacement velocity **V** of points described mathematically by a continuous and differentiable function of position (Gandar and Chalabi 1989; Silk 2006).

The continuity of **V** implies that values of the linear growth rate in different directions are coordinated and strongly depend on a direction (Erickson 1966). The relative elemental rate of growth in length at a given point along the direction **e**_s_ is defined by the equation (Hejnowicz and Romberger 1984): $R_{{1\left( {\text{s}} \right)}} = \left[ {\left( {\text{gradV}} \right) \cdot {\text{e}}_{\text{s}} } \right] \cdot {\text{e}}_{\text{s}}$ where **e**_s_ is the unit vector of the direction and dots represent scalar product. As that gradient acting on vector gives the second-rank operator (Spiegel 1959), the field of growth rates of the organ is of a tensor type (Silk and Erickson 1979; Kennaway et al. 2011). If **V** is known, such field can be determined with the aid of the growth tensor (GT, Hejnowicz and Romberger 1984) calculated from grad **V** (Supplementary material 1).

Modeling by use of the GT was used to study spatial and directional variation of growth rates in root apices (Hejnowicz 1989; Nakielski 1991; Hejnowicz and Karczewski 1993). It has appeared that in the root apex with the AC, there maximum volumetric growth rate occurs, which may relate to activity of the apical cell working as a single initial. Concerning directional *R*_l_ variation, locally three mutually orthogonal principal directions of growth (PDG) can be recognized unless growth is isotropic, as the directions in which *R*_l_ attains extreme values (maximal, minimal and of a saddle type). These PDGs, determined locally for many points, arrange themselves into three types of continuous lines called PDG trajectories (Hejnowicz 1984, 1989). Through every point, three such trajectories can be drawn. At the organ level, there is a pattern of PDG trajectories considered steady, if organ shape does not change in time. In roots (Nakielski 1987, 2008), similarly to other growing plant organs (Dumais and Kwiatkowska 2002; Kwiatkowska 2004), such pattern can be observed in the cell wall system. Two families of continuous and mutually orthogonal lines commonly used to describe cell walls seen in a longitudinal section, known as periclines and anticlines (Sachs 1887), have been postulated to represent PDG trajectories (Hejnowicz 1984). Hejnowicz (1989) hypothesized that cells divide with respect to PDGs, a division wall typically is perpendicular to one of three PDG at the site of its formation. The question arises whether regularity of cell pattern observed in roots with the apical cell as well as a highly specific division program that takes place in merophytes prior to differentiation may result from cell divisions oriented according to this rule.

On the basis of the GT concept, the 2D simulation model for growth in which cells divide with respect to PDGs was worked-out (Nakielski 1999; Nakielski and Hejnowicz 2003). An application of the model to the root apex with the quiescent center, exemplified by radish, gave interesting results (Nakielski 2008). The virtual root apex grew realistically, indicating that cell divisions with respect to PDGs, are needed to generate cell pattern which is preserved during growth.

The present paper shows a similar tensor-based model, but for the root apex with the apical cell. The model, implemented on the example of the *Azolla pinnata*, assumes that growth field of the root apex is of the tensor type and cells divide with respect to PDGs. Using simulation, one can see how new merophytes are formed and cell pattern of the root apex develops in time. The obtained results indicate a role of principal growth directions in generation and maintenance of the cell pattern typical for the root apex with the apical cell. The divisions with respect to PDGs appear essential not only for coordinated growth of the individual cells in merophytes but also to these merophytes in the organ as a whole. Both the formative and proliferative divisions in merophytes are oriented taking PDGs into account but their highly specific division program needs regulation at the cellular level.

## Materials and methods

### Root material

**Fig. 2:**
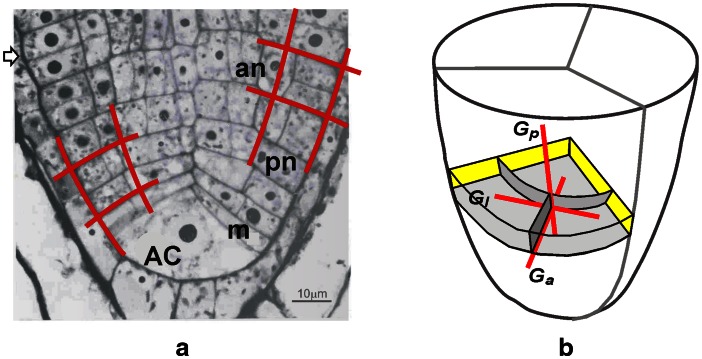
Cell pattern in the apical part of the *Azolla pinnata* root apex in which AC divided 43 times (**a**). Orientation of cell walls in the exemplary merophyte in 3D (**b**). **a** On the basis of the longitudinal section adopted from Gunning (1982) the exemplary periclines (pn) and anticlines (an) are shown. *AC* the apical cell, *M* youngest merophyte, root/cap junction (*arrowhead*). **b** Principal growth directions: *G*_p_, *G*_a_, *G*_l_ at the point of intersection of three cell walls are indicated, assuming the merophyte 5 from Fig. 1. The distal face of the merophyte (*light gray*) is perpendicular to *G*_p_, whereas longitudinal-tangential (*intermediate gray*) and longitudinal-radial (*dark gray*) walls are perpendicular to *G*_a_ and *G*_l_, respectively. The walls perpendicular to *G*_p_ and *G*_a_ generated in the model are described in this paper

Let us assume cell pattern in the axial section of *A. pinnata* root apex adopted from Gunning (1982) as representative for this species (Fig. 2a). In the cell pattern, all peculiarities of growth organization typical for the root apex with the single AC and merophytes can be seen. For our modeling, regularity of the cell wall system is the most important. Walls of cells located in different parts of the root apex manifest either periclinal or anticlinal orientation. Therefore, the cell pattern can be conveniently described by periclines and anticlines. Two periclines correspond to the root/cap junction, whereas borders between merophytes are represented by anticlines. Notice that every two mutually orthogonal cell walls locally tangent to particular pericline and anticline, though displaced as a result of continuous flow of cells from the distal portion of the root apex, retain their orthogonal intersections in time. This is because orthogonal intersections between both these clines are preserved during growth (Sachs 1887; Hejnowicz 1989). Orthogonal alignment of merophytes with respect to the root cap border as well as division walls resulting from longitudinal and transversal divisions within merophytes is evident. This indicates that peri-anticlinal arrangement of the cell wall system is especially important for *A. pinnata* root apex. If periclines and anticlines represent PDG trajectories (Hejnowicz 1984, 1989), regularity of the cell pattern may result from cell divisions oriented with respect to PDGs.

The cell walls in the exemplary merophyte are shown in 3D in Fig. 2b. Let us assume that these walls are formed with respect to PDGs in such a way that the merophyte lies in the plane defined by *G*_a_ and *G*_l_, i.e. it is perpendicular to *G*_p_, and two remaining walls resulting from longitudinal-tangential and longitudinal-radial divisions are perpendicular to *G*_a_ and *G*_l_, respectively. Notice that due to threefold symmetry of the root apex, in cell pattern, coming from the axial section, only two of three sectors can be seen. Moreover, the cell walls formed by the longitudinal-radial divisions (perpendicular to *G*_l_) are not represented in it. Accordingly, in our simulation model showing development of cell pattern in the axial plane, only cell divisions perpendicular to *G*_p_ (transversal, anticlinal in 2D) and *G*_a_ (longitudinal-tangential, periclinal in 2D) will be generated. To simplify the description, division walls perpendicular to *G*_p_ will be called anticlinal, whereas those perpendicular to *G*_a_ will be periclinal.

In regard to input data for computer simulation, the AC with the youngest merophytes and contacting cells of the root cap was assumed. They were digitized on the basis of Fig. 2a taking the merophyte corresponding to the stage 7 in Fig. 1 as the oldest one. Orientation of cell walls with respect to the root axis was estimated using published sections (Gunning et al. 1978; Gunning 1982) and our own anatomical study of *A. pinnata* root apices. In this study, serial sections of Steedman’s wax-embedded root tips were stained by the periodic acid-Schiff reaction and observed by brightfield and epifluorescence microscopy.

The 2D tensor-based simulation model for growth of *Azolla pinnata* root apex.

**Fig. 3:**
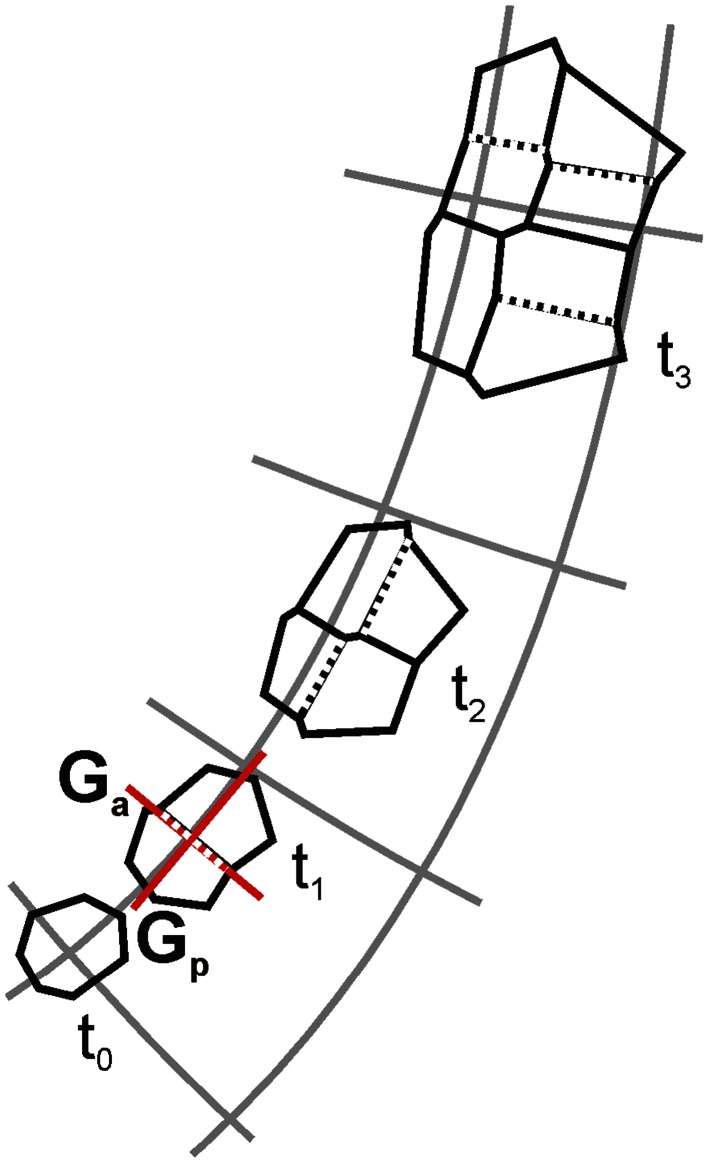
The computer-generated sequence illustrating algorithm of the 2D tensor-based model for growth in which cells divide with respect to PDGs, *gray lines* represent PDG trajectories. From the cell assumed at *t*_0_ the whole cell packet is obtained at *t*_3_; notice how the cell packet position changes with respect to the pattern of PDG trajectories. Cells enlarge and divide when critical value of their area is exceeded. The division wall can be perpendicular either to *G*_a_ or *G*_p_ (*red*) but the direction that gives division by the shorter wall is chosen, all newly formed walls are indicated by *dashed lines*. After formation, the new wall is diminished a little, causing a redefinition of angles in points of its attachment

To generate growth of *A. pinnata* root apex, the simulation model described previously (Nakielski 2008) was used. Briefly, the model is composed of three elements: the polygon meshwork representing cell pattern at the input, the GT field that generates growth, and the cell division algorithm according to which cells divide with respect to PDGs. The temporal sequences of the simulated growth are obtained by operational application of the GT field to the meshwork. During growth, the meshwork expands, deforms, and new cells are formed through divisions. The division occurs when the cell area assumed as critical is exceeded. Then the parent cell is replaced by two daughter cells separated by a division wall oriented with respect to one of PDGs. In the simulation plane that corresponds to the central longitudinal section there are two PDGs: *G*_a_ and *G*_p_. Previously, (Nakielski 2008) both these directions were considered, and finally, the PDG for which the division wall could be shorter was chosen (Fig. 3). In the present model, this rule is modified so that a choice of *G*_a_ or *G*_p_ depends on the cell state (will be described). After formation, the division wall is shortened slightly, with respect to its former length (Nakielski 2008), to have a more realistic three-way junction at each end of the new wall.

#### The meshwork

**Fig. 4:**
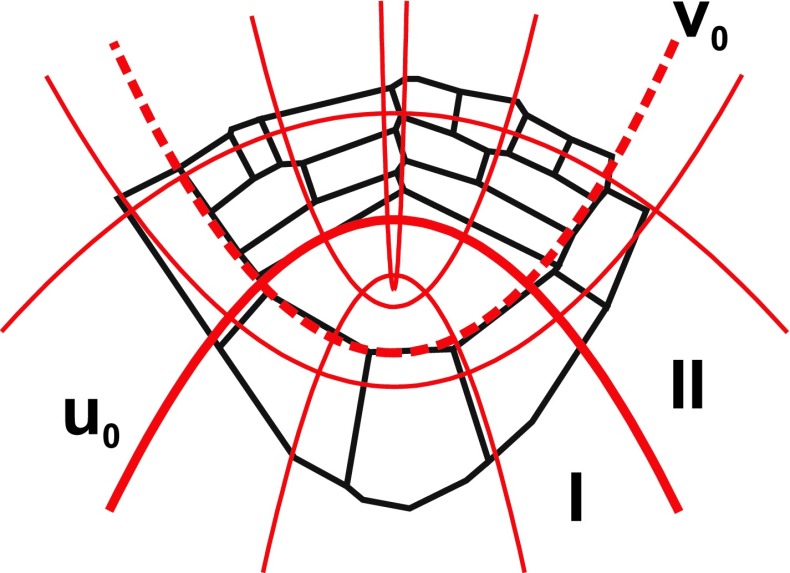
The polygon meshwork representing cell pattern in apical part of the *Azolla pinnata* root apex and the growth tensor field (*red*) applied to it. In the meshwork, cells are described by polygons. The growth field, defined in the paraboloidal coordinates (u, v, φ), is visualized by u and v lines which represent PDG trajectories. The field consists of two zones, I and II, separated by the line *u*_0_. Its application to the meshwork is such that assuming *v*_0_ = 0.42 as the root/cap border (*dashed*) the proximal faces of the apical cell are tangent to *u*_0_ = 0.44 (*thick*). Under this application, the zone I generates growth of the apical cell and distal part of the root cap, whereas the zone II—of merophytes and lateral part of the root cap

Individual cells were described by polygons and arranged into a meshwork (Nakielski 2008) in which two neighboring polygons had a common side, whereas three such polygons had a common vertex (three-way junction). At the start of the simulation, the meshwork consists of 19 cells (Fig. 4). It was an open structure in this sense that new walls, resulting from cell divisions, were added to it. At both ends of the division wall, new three-way junctions were formed. Due to shortening of the division wall, which was different depending on location, angles at each three-way junction were similar in that they were usually occurring in a given region of the root apex.

#### Growth tensor (GT) field

The displacement velocity field, **V**, defined by Hejnowicz (1989) was used. In the paraboloidal coordinate system (u, v, φ), the **V** vector is composed of three physical components: **V**_u_, **V**_v_ and **V**_φ_. Assuming the absence of rotation around the root axis during growth we have **V**_φ_ = 0, while the two remaining components are the following: ${\text{V}}_{\text{v}} = h_{\text{v}} \frac{{{\text{d}}v}}{{{\text{d}}t}}$, ${\text{V}}_{\text{u}} = h_{\text{u}} \frac{{{\text{d}}u}}{{{\text{d}}t}}$ where $h_{v} = h_{u} = \sqrt {u^{2} + v^{2} }$ are scale factors of the coordinate system. We deal with the axial section through the apex that corresponds to the plane φ = const. For such a plane, the **V** field is considered two-dimensional. It consists of two zones, I and II, separated by the line *u*_0_ (Fig. 4). These zones are responsible to generate growth in different parts of the root apex: zone I—the apical cell and central part of the root cap, zone II—merophytes with the remaining part of the root cap. At every point, including the line *u*_0_, the **V** is continuous. Two components of the displacement velocity are given by the equations (Hejnowicz 1989):

$${\text{Zone}}\,{\text{I}}\quad \frac{{{\text{d}}u}}{{{\text{d}}t}} = au\quad \frac{{{\text{d}}v}}{{{\text{d}}t}} = kv\left( {u_{0} - \frac{{u^{2} }}{{u_{0} }}} \right)$$

$${\text{Zone}}\,{\text{II}}\quad \frac{{{\text{d}}u}}{{{\text{d}}t}} = au_{0} + c\left( {u - u_{0} } \right)\quad \frac{{{\text{d}}v}}{{{\text{d}}t}} = - bv\left( {u - u_{0} } \right)$$

where a, b, c, k are parameters. Values of these parameters were the following: *a* = 0.012, *b* = 0.005, *c* = 0.07, *k* = 0.035. They were determined heuristically, by simulation, by taking cell pattern of the real root apex as reference. Let us take as the example the parameter *c*, influencing a thickness of merophyte at different age. The thickness increases linearly with a value of *c.* Different values of this parameter were tested and the best approximation of the rate was reached for *c* = 0.07. Values of the other parameters were specified in a similar way.

By having **V**, the tensor field of growth rates of the root apex was able to be determined. In correspondence to **V**, the field consisted of two zones; I and II. Figure 4 shows application of the field to the meshwork. The line *v*_0_ = 0.42 corresponded to the root/cap border, and the proximal faces of the apical cell were tangent to *u*_0_ = 0.44. Under such the application, slightly modified in comparison to previous ones (Hejnowicz 1989; Nakielski 1991), PDG trajectories correspond to lines of the coordinate system (see Supplementary material 1), especially in the region of a formation and development of merophytes. It was assumed that a pattern of the coordinate lines represents PDG trajectories in the axial plane of the root apex.

**Fig. 5:**
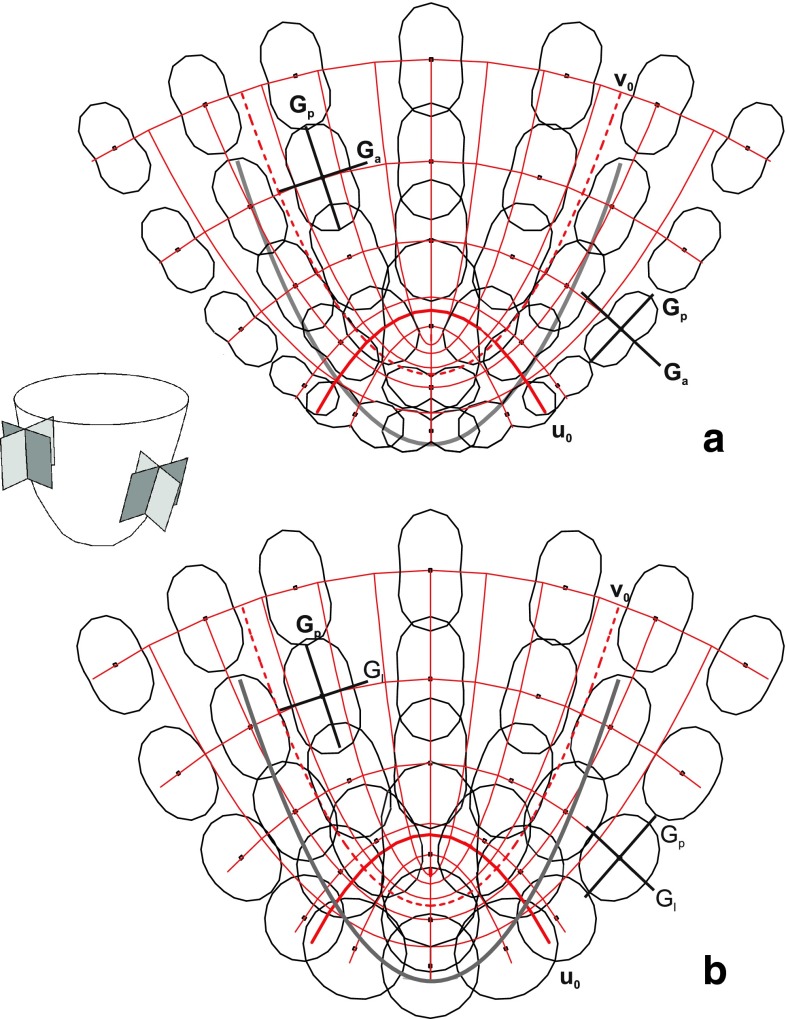
The growth field assumed to generate growth of the *A. pinnata* root apex. **a** Directional variation of the linear growth rate visualized in the axial plane. **b** As in (**a**), but for the tangential planes. The scheme on the left side shows orientation of both types of planes in 3D. At a given point value of the rate along a particular direction is proportional to the distance from the point to the plot surrounding this point. Thin *red lines* represent PDG trajectories, the *u*_0_ (*thick*) separates the zones I and II, the *v*_0_ (*dashed*) corresponds to the root/cap junction. The root cap border (*gray*) and two pairs of PDGs (*black*) at exemplary points are indicated: *G*_p_ and *G*_a_ in (**a**), and *G*_p_ and *G*_l_ in (**b**)

The spatial and directional variation of the linear growth rates (*R*_l_) within the root apex is shown in Fig. 5. The highest *R*_l_ values are in the central region and decrease with increasing distance from the tip in both periclinal and anticlinal directions. In terms of PDGs, everywhere, the highest growth rate is along *G*_p_ (periclinal direction), the rates along two remaining PDGs are significantly smaller. Comparing the rates along *G*_a_ (anticlinal direction) and *G*_l_ (latitudinal direction), it can be seen that they are similar at the root axis and reaching maximal rates in the central region. However, going from this region to the root tip, the rate in *G*_a_ decreases (Fig. 5a), and the rate in *G*_l_ remains relatively high (Fig. 5b). For that reason, at the root peripheries, the rates in *G*_a_ increase, whereas the rates in *G*_l_ decrease slightly with distance from the tip.

#### Algorithm for cell divisions

As mentioned earlier, cells divided with respect to PDGs and orientation of a division wall was determined by cell state. The apical cell could be in two states: 1 and 2. It divided always perpendicular to *G*_p_, however, in state 1, a new merophyte was produced to the sector on the left, whereas, in state 2—to the sector on the right. In every case, the division was unequal and the newly formed wall was tangent to *u*_0_. After division, the cell state was changed from 1 to 2 and vice versa.

**Fig. 6:**
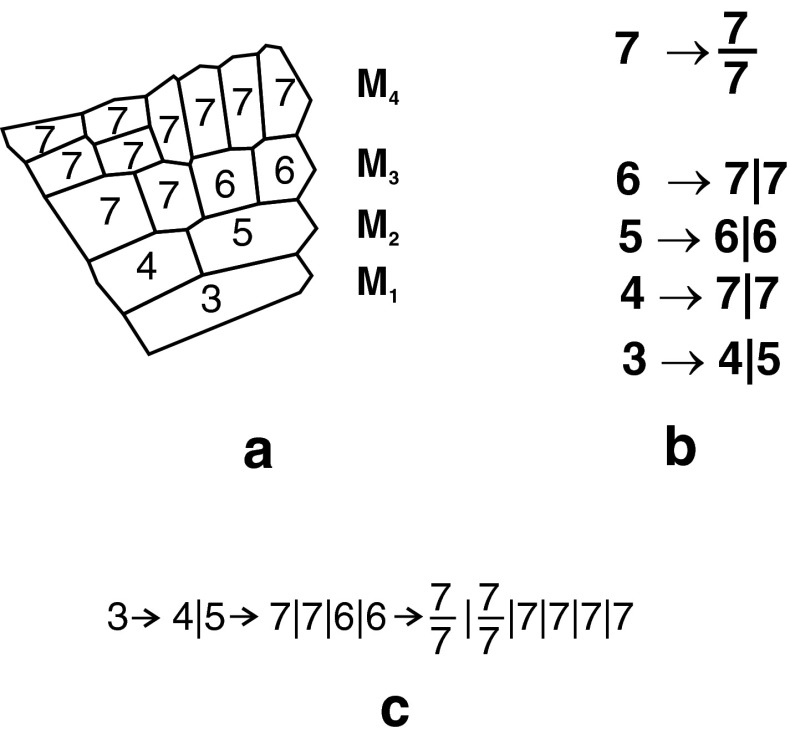
The cell state and their changes during development. **a** The states of cells in four successive merophytes. **b** Rules according to which the stages are changed by transversal (*dash*) and longitudinal-tangential (*vertical line*) cell divisions. **c** Notation showing development of merophytes from M_1_ to M_4_ in (**a**) described by the rules defined in (**b**). For further explanation, see text

The cell initiating merophyte was in state 3 and cells formed within the merophytes were in states 4–7 (Fig. 6a). How cell state affected cell division is shown in Fig. 6b. If the cell was in one of the states from 3 to 6, its division was perpendicular to *G*_a_ (longitudinal). The cells in states 3 and 6 divided into two equal parts, whereas division of cells in states 4 and 5 was unequal. The state 7 resulted in division perpendicular to *G*_p_ (transversal) and both daughter cells were equal. This state was final in the sense that both daughter cells and their derivatives were in the same state 7 as the parent cell.

Figure 6c shows changes in the state of cells during the merophyte development. The youngest merophyte (state 3) divides perpendicular to *G*_a_ producing cells in states 4 and 5. The cell at state 4, located closer to the root/cap border, divides perpendicular to *G*_a_ producing daughters in state 7. In further development, both daughters divide perpendicular to *G*_p_. The cell at state 5, in turn, divides unequally perpendicular to *G*_a_ giving two cells each in state 6. Their daughter cells reach state 7, which is the final state. Further divisions of these cells are perpendicular to *G*_p_. The root cap cells were once together in state 7 which means that they always divided transversally into two equal cells.

The cells of *A. pinnata* root apex differ a lot in their dimensions. For this reason, the area assumed as critical for cell division (*A*_cr_) was not the same for all cells. In general, it was at the level of 150 % of the mean cell area (*A*_av_). In estimation of *A*_av_, the apical cell, cells in merophytes and cells in the root cap were considered separately. Also, the shortening of division walls was not uniform at both ends of the new wall. For example, the wall cutting of new merophyte was diminished with respect to its former length about 1.2 % at the outer, and 7 % at the inner end. The shortening of new walls in merophytes depended on their orientation. Namely, the walls resulting from division perpendicular to *G*_a_ were shortened by 15 % and 8 % at the distal and proximal ends, whereas, divisions perpendicular to *G*_p_—by 8 % and 2 % at inner outer ends, respectively. The cells of the root cap were shortened by 1.2 % at both ends.

#### Simulations

**Fig. 7:**
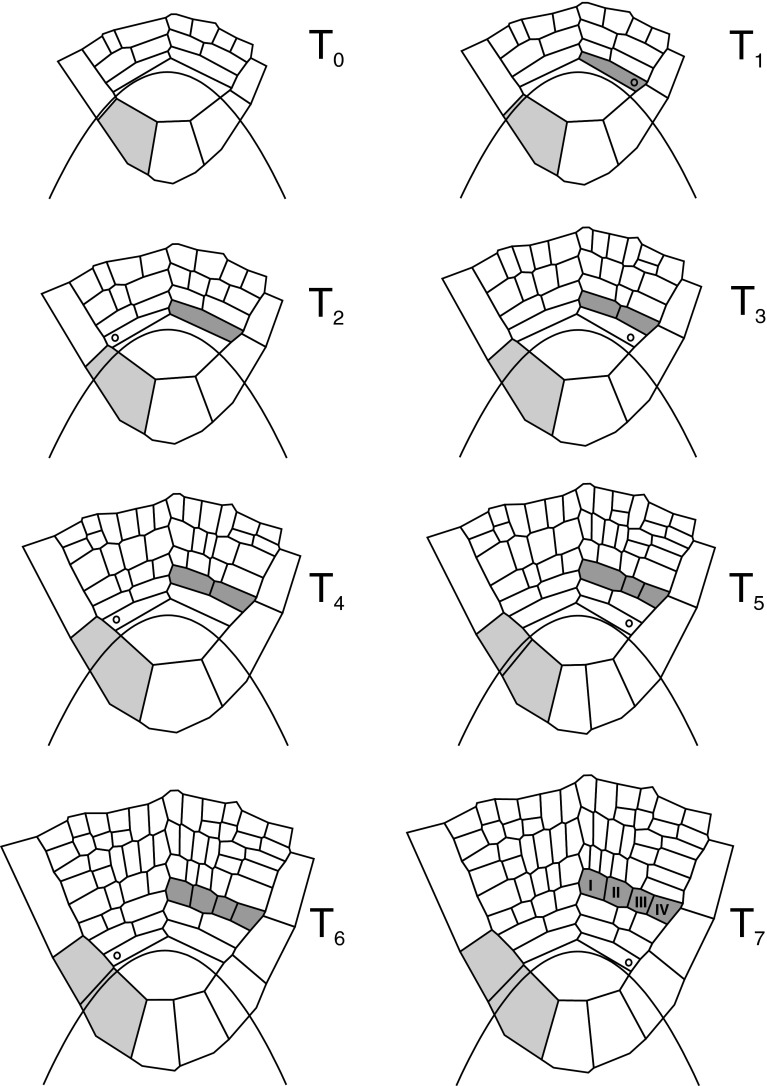
Computer-generated sequence of development of cell pattern in the *Azolla* root apex: times from *T*_0_ to *T*_7_. Under control of GT field, here, represented by the line *u*_0_, cells grow and divide with respect to PDGs. Newly formed walls are perpendicular either to *G*_p_ or *G*_a_ depending on their present state. Notice development of the exemplary merophyte (*dark gray*) and the root cap cell (*intermediate gray*). The newly formed merophytes are indicated (*open circle*); fates of the cell I–IV are considered in the text

**Fig. 8:**
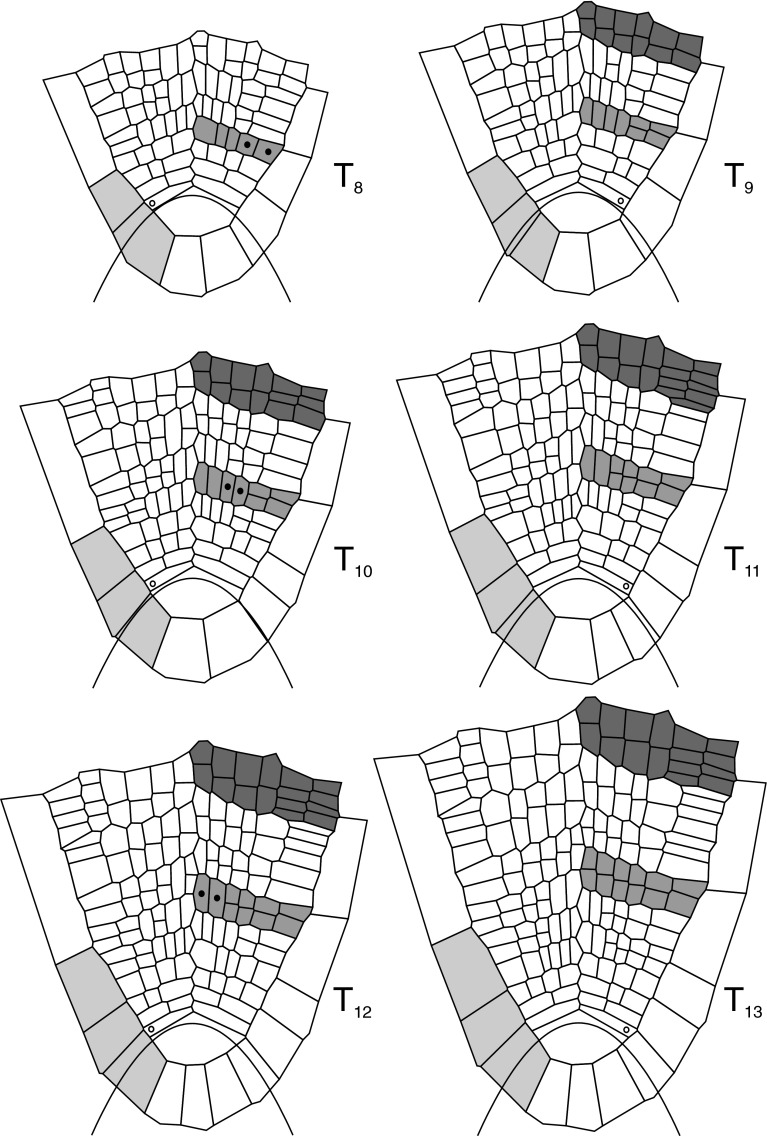
As in Fig. 7 but the times from *T*_8_ to *T*_13_. The cells marked by *closed circles* are precursors of the particular root tissues (for details see the text)

The simulation started from the meshwork is shown in Fig. 4. After ten steps, each with Δt = 0.02, the first loop (L_1_) was finished, and the meshwork returned to the input. Then the next loop (L_2_) was performed and the procedure was repeated. The whole simulation consisted of 16 loops during which the meshwork could be printed out at every step. The results presented in Figs. 7 and 8 show the meshwork in times corresponding to divisions of the apical cell, labeled with *T*_i_. In particular, the times *T*_1_ and *T*_13_ represent the meshwork after first and last division of the AC which occurred in L_3_ and L_15_, respectively.

The GT field was steady by definition. At *T*_0_, its application to the meshwork is as in Fig. 4, i.e. the root/cap border coincided with the line v_0_ = 0.42. However, in the course of the simulation, the border was slightly displaced proximally with respect to this line (by a distance not greater than 1 % of the apical cell height). It returned to the previous position at the start of each new loop.

### Statistical analysis

Statistical analysis was performed to estimate differences in orientation of the newly formed walls between real and virtual root apices. Cell walls coming from nearly central sections of six roots (both sectors) were taken into account; four coming from our own studies and two published by Gunning (1982). For every root apex, the angle between the wall and the root axis was estimated by division into three classes of cell walls: A—cutting off new merophyte, B, C—resulting from longitudinal-tangential (B) and transversal (C) divisions of cells in merophyte. For each class, at first, differences in angles between particular roots were analyzed using the ANOVA test. Then the samples coming from real roots, taken together for a given class, were compared with corresponding data obtained for a single virtual root in course of the whole simulation. In this case, the *t* test for independent samples was applied.

Also, the width ratio of two neighboring merophytes in the same sector (older to younger, i.e. M_i+1_/M_i_, in Fig. 6a) was estimated in both sectors of real and virtual roots. The width was measured in the central part of a merophyte. Assuming that the corresponding merophytes M_i_ of real and virtual roots make pairs, the *t* test for dependent samples was used. All tests were performed using STATISTICA, version 10, at the significance level 0.05.

## Results

The computer sequence of growth and cell divisions in the root apex of *A. pinnata* are shown in Figs. 7, 8 and Supplementary material 2 (video file). The cell pattern assumed at the input expands from *T*_0_ to *T*_7_ in Fig. 7 and from *T*_8_ to *T*_13_ in Fig. 8. In the course of the whole simulation, the apical cell divided 13 times and the total number of cells in the meshwork increased from 19 to more than 170. At every time-step, the GT field operated in such a way that the apical cell remained under control of the zone I, whereas merophytes developed in the zone II. Cell divisions were perpendicular to *G*_a_ (longitudinal-tangent division, periclinal in 2D) and perpendicular to *G*_p_ (transverse division, anticlinal in 2D).

New merophytes are cut off perpendicular to *G*_p_, alternately to the left and right side of the root apex (Figs. 7, 8). The first new merophyte is formed at *T*_1_ on the right side (light gray in Fig. 7). Let us consider development of this merophyte during the whole simulation. At *T*_2_, the cell giving rise to the merophyte increases in area and at *T*_3_, divides longitudinally (perpendicular to *G*_a_). Two daughters cells are obtained; the inner lying closer to the root axis and the outer that keeps contact with the root/cap border. From *T*_3_ to *T*_4_, both cells expand and then divide longitudinally as the parent cell at *T*_5_ and *T*_6_. Later, at *T*_8_ and *T*_9_ (Fig. 8), the next longitudinal division occurs in two inner cells. At the same time, starting from *T*_9_, the outer cells divide transversally (through division perpendicular to *G*_p_) and such division is reached by the innermost cells of the merophytes at *T*_13_. In reference to Gunning’s (1982) results, it is possible to indicate precursors of particular tissues. Using notation of the cell rows I–IV adopted from Fig. 1 (merophyte 7), they have been established as follows (Fig. 8): at *T*_8_—for the epidermis and root hairs (row IV) and outer cortex (row III), at *T*_10_—for the inner cortex and endodermis (row II), and at *T*_12_—for the pericycle, xylem, sieve elements, phloem parenchyma (row I).

Notice that at the end of the simulation (*T*_13_ in Fig. 8), the considered merophyte (light gray) appears to be similar to the younger merophyte marked as dark gray at *T*_9_ in Fig. 7. Hence, it is possible to predict a sequence of divisions of cells of the dark gray merophyte. Namely, during a period from *T*_9_ to *T*_13_ (Fig. 8) cells of this merophyte will expand and new transverse divisions will occur in the outermost cell. Further divisions of cells of this merophyte will be of a proliferative type.

Both analyzed merophytes are situated on the left side of the root axis, but it is easy to notice that merophytes on the opposite side developed in a similar way. Now let us focus our attention on the root cap. There are a few cell divisions in this region occurring only in the inner cell layer. As assumed, all these divisions are perpendicular to *G*_p_. Notice the cell at *T*_0_ located in zone 1 (gray in Fig. 7). This cell expands periclinally and divides anticlinally at *T*_5_ giving two daughter cells. Since the division wall happens to be near the border between the zones 1 and 2, these daughter cells find their way to different zones and their fates are different. The one controlled by zone 2 expands without division and is displaced proximally into the root peripheries whereas the other one remaining in zone 1 grows and divides as its parent cell at *T*_9_.

**Fig. 9:**
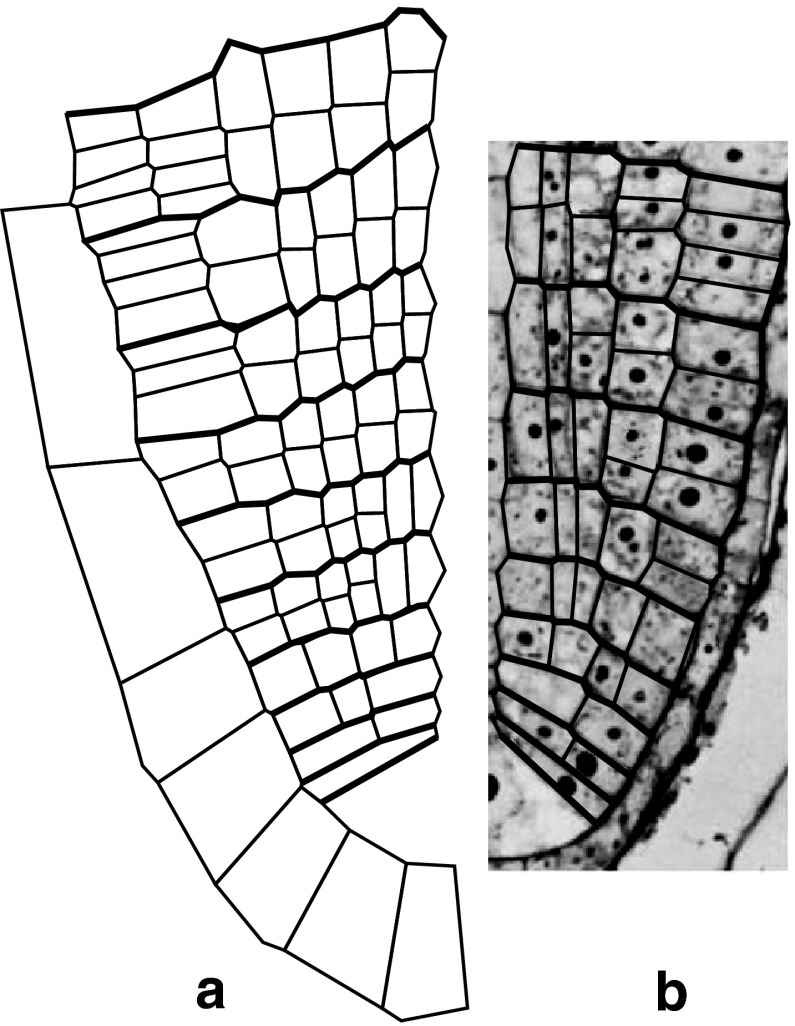
Comparison of cell pattern of the virtual and real *Azolla pinnata* root apex (one sector only). **a** Ten virtual merophytes come from the model presented in this paper (*right* side of the root apex at *T*_13_ in Fig. 8). **b** The root section including nine merophytes comes from Gunning et al. (1978). The simulated cell pattern is realistic as far as dimension of successive merophytes and orientation new walls resulting from divisions of cell within the merophytes are concerned

Cell patterns of both real and virtual root apices are compared in Fig. 9. It can be seen that the computer-generated cell pattern is generally similar to that observed in the real root apex, especially concerning longitudinal dimension of successive merophytes as well as orientation of formative and proliferative divisions in the merophytes. Small discrepancies between the real and modeled roots relate to number cells in merophytes and not perfectly anticlinal shape of the merophytes generated in the model.

The statistical analysis of orientation of cell walls with respect to the root axis gave the following results. In all considered classes (A, B, C), the ANOVA test provided the absence of significant differences in orientation of corresponding walls between considered real roots; for each class, the obtained *P* values are much greater than 0.05 (A 0.99, B 0.88, and C 0.63). The *t* test has shown the absence of statistically significant differences in orientation of cell walls between corresponding samples in real and virtual roots (Table 1). The ratio taken from width of neighboring merophytes decreased with the merophyte age in both real and virtual roots (Table 2). The *t* test has shown the absence of statistically significant differences in ratio between corresponding samples of real and virtual roots (Table 3). This means that virtual and real roots are also statistically similar, at least taking considered characteristics into account.

**Table 1:** Orientation of newly formed walls in real and virtual root apices

	Real roots		Virtual root		*t* test	
	*n*	x_av_ ± SD	*n*	x_av_ ± SD	*t*-value	*P*
A	36	58.06 ± 5.09	12	60.17 ± 2.08	1.39	0.17
B	36	16.31 ± 6.07	57	14.11 ± 6.30	1.66	0.10
C	36	81.36 ± 7.99	48	78.71 ± 7.26	1.59	0.12

**A** The walls cutting off new merophyte. The walls resulting from formative (**B**) and proliferative (**C**) divisions in merophytes. The angle between the new wall and the root axis was measured in degrees

*n* number of walls, *x*_*av*_ mean value, *SD* standard deviation

**Table 2:** The ratio of width of two neighboring merophytes obtained for virtual (second column) and real (third column) *Azolla* root apices

Merophytes	Virtual root	Real root
2:1	1.27	1.28
3:2	1.29	1.30
4:3	1.29	1.28
5:4	1.21	1.23
6:5	1.22	1.20
7:6	1.16	1.18
8:7	1.14	1.16
9:8	1.14	1.12

The first column indicates from which merophytes the ratio was taken. The third column shows mean values

**Table 3:** Comparison of the ratio width of two neighboring merophytes in the real and virtual *Azolla* root apices

	Real root		Virtual root		t test	
	*n*	x_av_ ± SD	*n*	x_av_ ± SD	*t*-value	*P*
Ratio	31	1.25 ± 0.12	31	1.21 ± 0.07	1.81	0.07

The samples were dependent and every ratio of the virtual apex was paired with corresponding ratio in the real apex

*n* number of walls, *x*_*av*_ mean value, *SD* standard deviation

**Fig. 10:**
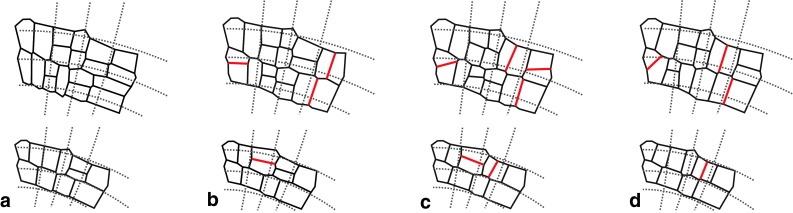
The computer-generated cell pattern in two merophytes obtained by use of different division rules. **a** Cell divides with respect to PDGs with the algorithm presented in this paper. **b** As in (**a**) but not taking cell states into account. **c** Cell divides perpendicularly to the nearest wall (Sach’s hypothesis). **d** Cell divides by the shortest path (Errera’s hypothesis). The merophytes at the bottom and top correspond to the oldest ones at *T*_2_ and *T*_6_ in Fig. 7, respectively. In **b**-**d**, the division walls with orientation different than in (**a**) are indicated (*red*)

In our model, fates of individual cells are determined by their position in GT field. Accordingly, PDGs at a given position affect both cell extension and cell division. What happens when other rules for cell divisions (Besson and Dumais 2011; Müller 2012), are used to simulate the development of exemplary merophytes can be seen in Fig. 10. As shown in Fig. 10b, if cells divide with respect to PDGs but their states are not taken into account, in some cells, longitudinal divisions instead transversal ones and vice versa (transversal instead longitudinal) occur. This causes that realistic cell division sequence, generated in Fig. 10a, to be disturbed. In the case of two remaining rules that implement Sach’s and Errera’s hypothesis, a similar disturbance between longitudinal and transversal divisions takes place (Fig. 10c, d). However, apart from this there are oblique division walls not observed in merophytes of real roots. We conclude that only the rule in which both the PDGs and the cell states are taken into account results in realistic cell pattern with realistic cell division program in merophytes.

## Discussion

This paper presents a 2D model of growth and cell division in the root apex with a single apical cell. The model shows development of cell pattern in the central longitudinal section of the root apex, assuming that growth field of the organ is of a tensor type (Hejnowicz and Romberger 1984) and cells divide with respect to principal directions of growth (PDGs). How PDGs affect cellular pattern of the root apex with the quiescent center was described previously (Nakielski and Hejnowicz 2003; Nakielski 2008). The present paper is the first in which a tensor field of growth rates has been used to generate growth together with cell divisions in the root apex with the apical cell and merophytes.

The root apex of *Azolla pinnata* root is predisposed to such modeling. It has relatively simple but precise construction (Gunning et al. 1978; Gunning 1981) and successive divisions of the AC and its derivatives are the most completely documented among ferns. Moreover, cell pattern of the root apex derives from activity of one cell working as a single initial. A remarkable regularity of the cell pattern may suggest some special properties of the AC (Gifford 1983; Barlow 1994). However, our simulations indicate an important role of principal growth directions; the cell pattern is regular and becomes steady due to cell divisions oriented with respect to PDGs. New merophytes are formed perpendicularly to the *G*_p_, whereas division walls in merophytes are formed perpendicularly to *G*_a_ and *G*_p_ (longitudinal-tangential and transversal divisions, respectively). Adopting growth rate anisotropy is shown in Fig. 5, the transversal divisions are perpendicular, whereas longitudinal ones are tangent to the direction of maximal *R*_l_. The realistic development of cell pattern of the virtual root apex may suggest that the apical cell and its derivatives are able to detect PDGs at a site of their location and orient division walls with respect to them.

The PDGs are an integral part of a growth field (Hejnowicz and Romberger 1984). In our model they are considered locally and at the organ level through the PDG field. Locally they affect cell expansion and cell divisions (orientation of division wall), whereas at the organ level, the stability of the root shape and coordinated development of merophytes within the apex (cell fates depend on PDGs via position in GT field) are affected. As the PDG pattern determines the orientation of volume increase and is predetermined by the existing structure of the growing body (Hejnowicz and Hejnowicz 1991), there is a feedback between growth field and the cell wall system. When the volume increase is accompanied with proliferation, cell divisions are involved in mechanism governing growth and formation of division wall with respect to PDGs, which is natural. In the broader meaning, PDGs are needed for coordinated growth of individual cells and the organ as a whole.

Concerning orientation of the division wall, several hypotheses have been proposed (reviewed by Smith 2001; Müller 2012; Minc and Piel 2012). It was postulated that cells divide: perpendicular to the main axis of growth (Hofmeister 1863), nearly perpendicular to the existing walls (Sachs 1878, 1887) and along the shortest path, dividing the cell into two equally sized daughters (Errera 1888). These geometrical hypotheses emphasize local properties of dividing cells, not taking organ level and directional signals into account. Besides, they are useful mostly for isotropic growth (Sahlin and Jönsson 2010) and geometrically uniform cells (Besson and Dumais 2011; Alim et al. 2012). Asymmetric divisions have been recently used to model stomata lineages in leaf epidermis through a polarity-switching mechanism (Robinson et al. 2011). Also mechanical stress has been suggested to affect orientation of division plane (Green 1980; Lintilhac and Vesecky 1984), receiving support in empirical observations (Lynch and Lintilhac 1997; Hamant et al. 2008; Potocka et al. 2011). The Hejnowicz’s hypothesis, according to which cells divide with respect to PDGs, combines geometrical and mechanical approaches. On the one hand, it can be seen as generalization of the cell geometry based rules (Hejnowicz and Hejnowicz 1991). On the other hand, its relation to mechanical stress is clear because from the point of view of mechanics, growth is an irreversible deformation that results from stresses in the cell wall system (Nakielski and Hejnowicz 2003). Both the growth rate and the mechanical stress are tensor quantities and one can expect a relationship between their principal directions. If mechanical cues are sensed and interpreted by ‘highly dynamic and regulated microtubule cytoskeleton (Mirabet et al. 2011) they may give rise to PDG oriented cell divisions via the stress. The mentioned relationship needs investigations, however, empirical data suggest (Alim et al. 2012) that cell division is parallel to the direction of maximal stress. By analogy of previous modeling (Nakielski 2008), such situation may take place in the case of proliferative divisions in merophytes.

The rule according to which cells divide with respect to PDGs is primary for the present model. The majority of remaining rules are used mainly to ‘translate’ 3D cellular architecture and cell division sequence which occurred in the real root apex (under threefold symmetry about central longitudinal axis), to our 2D model in which merophytes are produced only to two sectors and to the left and right. In addition, to be as close as possible to empirical data, these remaining rules are numerous and deal with details. This creates the impression of “hard-wiring” a correct division pattern by which the primary role of PDGs is obscure. In this context, it is worth noticing that if divisions of the apical cell are considered in 3D, the rule about the AC state becomes unnecessary because all merophytes are formed exactly in the same way as perpendicular to *G*_p_ at the site of its formation. The helical formation of successive merophytes may be a consequence of geometrical factors resulting from the division parallel to proximal face giving rise to the first merophyte of the new root. Whether they are produced clockwise or counterclockwise may depend on random factors as that both are similarly frequent either in root (Gunning 1982; Korn 1993) or shoot (Imaichi 1988) apices.

The computer-generated cell pattern was realistic but also some discrepancies between real and virtual roots, related to number of cells and not perfectly anticlinal alignment of merophytes were observed (Fig. 9). Such discrepancies seem to be inevitable when a highly specific cell division program designated for three sectors has been applied to two sectors and under limitation to the longitudinal section. In addition, our attention was focused rather on the order of formative and proliferative divisions in merophytes, not cell pattern in particular sectors of the real apex. Also the simplification according to which cell walls are represented by straight lines is important; in the case of the AC, it is responsible for not exactly anticlinal alignment of merophytes. On the other hand, even working with the microscopic section regarded as the best in the literature, it is worth noting that the section is not perfectly central (Gunning 1982) and two sectors, due to threefold symmetry of the root apex, are not equally represented in it.

The *Azolla pinnata* root growth is determinate (Gunning et al. 1978). The apical cell divides in sum 50–55 times but after approximately 35 divisions its mitotic activity is successively reduced and finally ceases when the root reaches the length of about 8–10 mm (Gunning et al. 1978). A similar reduction is also observed in roots of other species (Chiang and Gifford 1971; Nitayangkura et al. 1980; Gifford 1983). In our simulation, the AC divided 6–7 times per sector which corresponds to about 2/5 of the total number of its divisions. Such time period was sufficient to analyze the cell pattern maintenance, but too short to assume changes in a cell division rate. Such changes can be taken into account in future work focused on a relationship between the rate of growth and the rate of cell divisions during whole period of mitotic activity of the AC.

In the present modeling the mature root apex is considered. The question arises how such cell pattern is formed. The problem has been already studied by use of the growth tensor (Hejnowicz and Hejnowicz 1991). The GT field, similar to the one considered here but of an unsteady type, was applied to the non-differentiated orthogonal grid of points. Under this field, the grid extended, deformed and a clear protrusion was formed. Adjusting by hand the apical cell, it was possible to observe deformation of this cell and provide for its divisions with respect to PDGs. Three routes of development, differing in orientation of the first division, were considered. They lead optionally to the root apex with a single and two apical cells (two routes). Adopting these results to the *A. pinnata* root apex, one can conclude that the first division of the apical cell (parallel to the distal face) is perpendicular to *G*_a_, whereas all next divisions parallel to each of three proximal faces, are perpendicular to *G*_p_.

Roots with a single apical cell and roots with a quiescent center differ in growth organization and in cellular architecture but are similar in PDG pattern (Hejnowicz and Karczewski 1993). This suggests that the tensor-based control related to PDGs is needed regardless of cellular organization of the root apex (quiescent center or the apical cell). It is worth mentioning that directional information included in PDGs is essential in the present paper to describe cell pattern maintenance during steady growth, and is also of importance for unsteady growth which takes place, for example, during the lateral root formation (Szymanowska-Pułka and Nakielski 2010).

The rule governing the cell state change is similar to ‘production rules’ commonly used in L-systems (Prusinkiewicz 1998, 2004). In the 2D modeling of growth of *Microsorium linguaeforme* gametophyte (de Boer and de Does 1990) cell divisions oriented periclinally and anticlinally were generated. However, such orientation did not result from PDGs and the tensor basis of the symplastic growth but only from the gametophyte geometry; periclinal division was tangent, whereas anticlinal-perpendicular to the organ surface. This cannot be surprising, because the plane tangent to the surface is defined by two PDGs: *G*_p_ and *G*_l_. Hence, periclinal and anticlinal divisions seen in a longitudinal section through the organ are in 3D perpendicular to *G*_a_ and *G*_p_ respectively, i.e. exactly as in the model presented here.

The L-systems appeared useful first of all to analyze genealogy of cells (Lück et al. 1988, 1994a, b; Barlow 1991; Lück and Lück 1993). In this aspect both shoot and root apices possessing one or more apical cells were investigated (Barlow et al. 2000, 2001). Such analysis does not need simulation performed at the organ level. Therefore, the present paper is the first in which development of cell pattern in the section of the whole root apex with the apical cell has been shown. It is also the first in which growth and divisions of individual cell are controlled on the one hand at the organ level, by growth field with PDGs, and on the other hand locally by factors influenced the cell state, type of cell division (perpendicular to *G*_p_ or *G*_a_, equal or not) and shortening of a division wall. Moreover, the present approach, though restricted to the case of a single apical cell, can be relatively easily adapted to describe growth in roots or shoots of *Azolla filiculoides* (Gifford and Polito 1981; Nitayangkura et al. 1980), *Equisetum* (Gifford and Kurth 1982; Gifford 1993) and *Ceratopteris richardii* (Hou and Hill 2002, 2004). The case with more than one apical cell requires deeper modifications.

## Electronic supplementary material

Below is the link to the electronic supplementary material.

Supplementary material 1 (DOCX 21662 kb)

Supplementary material 2 (MPG 5014 kb)

## Abbreviations

AC: Apical cell
GT: Growth tensor
PDG: Principal direction of growth
R_l_: Relative elemental rate of linear growth
