# Supplementary material for: The simulation model of growth and cell divisions for the root apex with an apical cell in application to Azolla pinnata
Source: Planta. 2013 Aug 30;238(6):1051–64. doi: 10.1007/s00425-013-1950-9 (PMC3898529; doi:10.1007/s00425-013-1950-9)
Supplement: Supplementary file 1 — Supplementary material 1 (DOCX 21662 kb) [file 425_2013_1950_MOESM1_ESM.docx]

# The simulation model of growth and cell divisions for the root apex with an apical cell

# in application to *Azolla pinnata* .

# Planta

# Anna Piekarska-Stachowiak, Jerzy Nakielski

Department of Biophysics and Morphogenesis of Plants, University of Silesia, Katowice, Poland

[anna.piekarska@us.edu.pl](mailto:anna.piekarska@us.edu.pl)

**The Growth Tensor and calculation of the linear growth rate**

**in paraboloidal coordinate system**

The symplastically growing organ is characterized by the continuous field of the displacement velocity (**V**) from which the growth tensor (GT) is calculated. The GT follows directly from the definition of the relative elemental rate of growth (R_l_) (Silk and Erickson 1979), namely R_l_ in a given direction **e_s_** can be expressed as follows (Hejnowicz and Romberger 1984):

$$R_{l(s)}=\lim_{\begin{aligned} \Delta t\to0 \\ \Delta s\to0 \end{aligned}} \frac{\Delta\left( \Delta s \right)}{\Delta s\Delta t}=\frac{d\frac{\mathrm{ds}}{\mathrm{dt}}}{\mathrm{ds}}=\frac{dV_{s}}{\mathrm{ds}}=\frac{d\left( \mathbf{V}\cdot\mathbf{e}_{s} \right)}{\mathrm{ds}}=\nabla\left( \mathbf{V}\cdot\mathbf{e}_{s} \right)\cdot\mathbf{e}_{s}=\left[ \left( \nabla\mathbf{V} \right)\cdot\mathbf{e}_{s}+\left( \nabla\mathbf{e}_{s} \right)\cdot\mathbf{V} \right]\cdot\mathbf{e}_{s}=\left( \nabla\mathbf{V} \right)\cdot\mathbf{e}_{s}\cdot\mathbf{e}_{s}=\left( \frac{\partial V_{p}}{\partial x_{q}}t_{p}t_{q} \right)\cdot\mathbf{e}_{s}\cdot\mathbf{e}_{s}=\left( T_{\mathrm{pq}}t_{p}t_{q} \right)\cdot\mathbf{e}_{s}\cdot\mathbf{e}_{s}=T_{\mathrm{pq}}e_{p}e_{q},$$

where **e**_s_ is the unit vector in direction s, **V** is the displacement velocity,$\nabla\mathbf{V}$ is dyadic which corresponds to the growth tensor T_pq_ in physical components and, using the coordinate system with coordinates denoted by p,q and summation convention, e_p_ is the component of **e**_s_ along p, t_p_ is the unit vector tangent to coordinate line p.

The GT generates the field of growth rates in the organ. Inherent characteristics of this field are principal directions of growth, PDGs (Hejnowicz and Romberger 1984, Hejnowicz 1984). At a given point are three mutually orthogonal PDGs unless growth is isotropic. These directions are called: periclinal, anticlinal and latitudinal. In two of them R_l(s)_ attains extreme values and the third one is perpendicular to the mentioned two. Between successive points of the field, PDGs change in a continuous way giving a pattern of PDG trajectories. Through every point passes three PDGs and three trajectories of PDGs. Each pair of PDGs defines a principal plane, each pair of PDG trajectories defines a principal surface. If GT field is maintained over time, the PDG trajectories are steady and can be approximated by lines of a natural curvilinear coordinate system (Hejnowicz, 1984). In this system, in the definition of R_l(s)_ developed above represented by the coordinates p,q, both **V** and GT have a relatively simple form.

A convenient natural coordinate system to generate GT field for the root apex that has a rotational symmetry appears to be a paraboloidal one (u,v,ϕ).


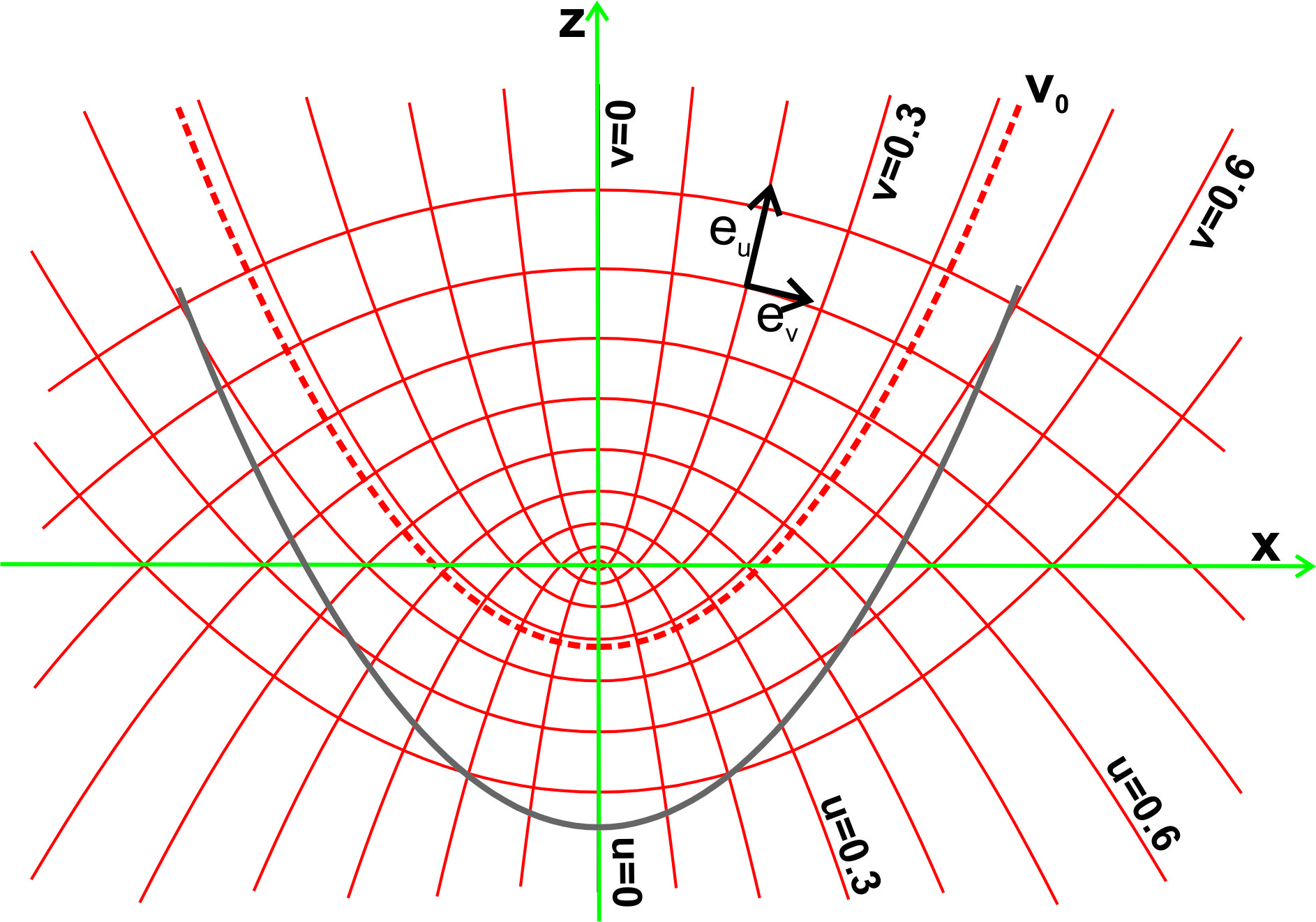


If the apex is considered in the axial section for which ϕ=0 and the root axis corresponds with z line of the rectangular system, as in the enclosed figure, we have: x=uv and z=0.5(u^2^-v^2^).

The system is of a confocal type. The focus located at the root axis mimics the focus of the pattern of peri- and anticlines of the real root apex. Assuming **V**_ϕ_=0, there are only two physical components of a displacement velocity vector: **V**_u_ and **V**_v_ which are given by the equations:

$$\mathbf{V}_{u}=\sqrt{u^{2}+v^{2}}\frac{\mathrm{du}}{\mathrm{dt}} \mathbf{V}_{v}=\sqrt{u^{2}+v^{2}}\frac{\mathrm{dv}}{\mathrm{dt}}$$

where $\frac{\mathrm{du}}{\mathrm{dt}}$ and $\frac{\mathrm{dv}}{\mathrm{dt}}$ are function of u and v.

The general form of the GT represented by physical components in the paraboloidal coordinates is:

$$T_{\mathrm{pq}}=\left| \begin{matrix} T_{\mathrm{uu}} & T_{\mathrm{uv}} & T_{u\varphi} \\ T_{\mathrm{vu}} & T_{\mathrm{vv}} & T_{v\varphi} \\ T_{\varphi u} & T_{\varphi v} & T_{\varphi\varphi} \end{matrix} \right|=\frac{1}{\sqrt{u^{2}+v^{2}}}\times\left| \begin{matrix} \frac{\partial V_{u}}{\partial u}+\frac{vV_{v}}{u^{2}+v^{2}} & \frac{\partial V_{u}}{\partial u}-\frac{vV_{u}}{u^{2}+v^{2}} & 0 \\ \frac{\partial V_{u}}{\partial v}-\frac{uV_{v}}{u^{2}+v^{2}} & \frac{\partial V_{v}}{\partial v}+\frac{uV_{u}}{u^{2}+v^{2}} & 0 \\ 0 & 0 & \frac{V_{u}}{u}+\frac{V_{v}}{v} \end{matrix} \right|$$

According to the definition of the linear growth rate the R_l_ in a considered direction α is obtained by multiplying twice the GT in dyadic form by the unit vector **e**_s_ in this direction:

$R_{l\left( \alpha\right)}=\sum\left( T_{\mathrm{ij}}t_{i}t_{j} \right)\cdot\mathbf{e}_{s}\mathbf{e}_{s}$.

In the paraboloidal system with ϕ=const. the unit vector of **e_s_** defined which reference to the tangent to the coordinate line u and v is $\mathbf{e}_{s}=t_{u}cos\alpha+t_{v}sin\alpha$, where t_u_ is the unit vector along the coordinate line u (v=const.), and t_v_ is the unit vector along the coordinate line v (u=const) at the considered point. Introducing **e_s_** into the equation for R_l_, we obtain:

$R_{l(\alpha)}=T_{\mathrm{uu}}\cos^{2}\alpha+T_{\mathrm{vv}}\sin^{2}\alpha+\left( T_{\mathrm{uv}}+T_{\mathrm{vu}} \right)sin\alpha cos\alpha$.

The principal directions of the GT are defined as the directions in which the R_l_ attain extreme values. In terms of the above equation the directions are specified by the angles α_1_, α_2_ between PDGs and the tangent to coordinate line u (v=const.) at the considered point. Namely, along the principal directions we have: $d(R_{l(s)})/d\alpha=0$. By solving this equation, locally two values for α, i.e. the two principal directions in the axial plane can be obtained. One direction is periclinal-longitudinal, the second is anticlinal. The third principal direction, perpendicular to the plane ϕ=const.; is periclinal-tangential. Using this approach for many points, PDG trajectories at the organ level were drawn (Hejnowicz, 1989), in particular, on the example of the root apex with the apical cell (AC), without application to any species.

In modelling of growth in shoot and root apices, PDG trajectories should be adjusted to the pattern of periclines and anticlines observed in their axial sections. Moreover, the grid formed by point of the virtual organ should preserve this pattern during growth. In practice, these trajectories are either drawn by a computer using the approach described above, or approximated by lines of the coordinate system assumed as natural one. Below, the results of application of both ways to the root apex of Azolla pinnata are shown, assuming v_0_=0.42 as the root/cap border (grey), and u_0_=0.44 (red) as the proximal limit of the AC. In (a), the PDG trajectories are redrawn from Hejnowicz (1989), in (b), they are represented by the u and v lines of the paraboloidal system.


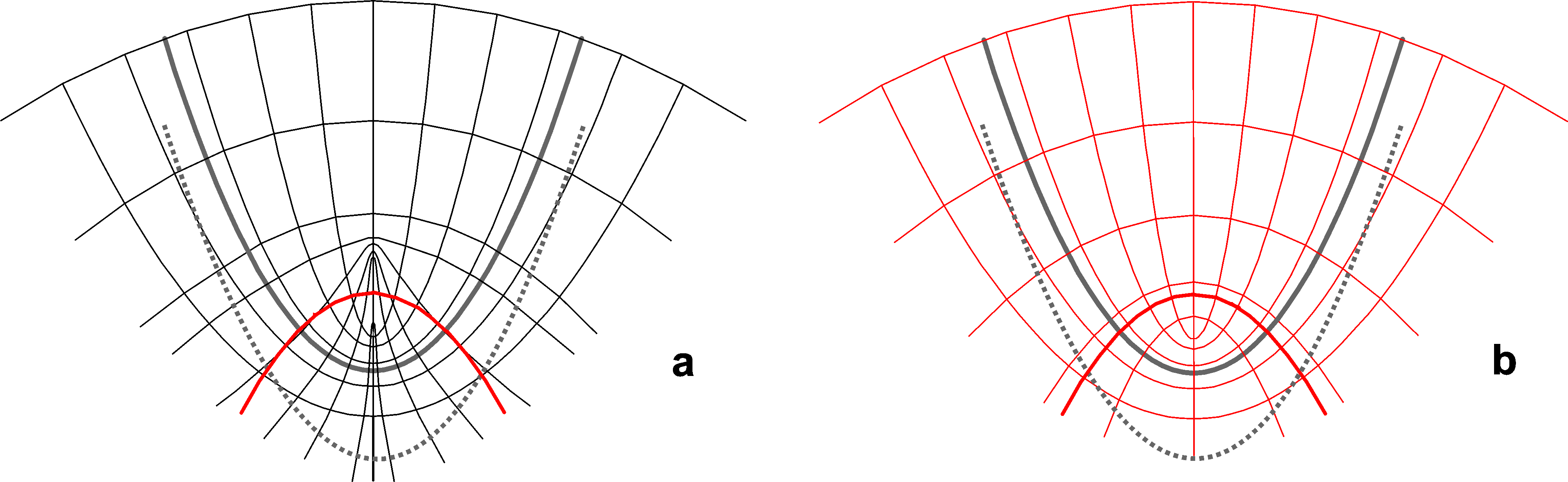


Though both patterns were obtained using slightly different values of parameters specifying du/dt and dv/dt, the PDG trajectories are generally similar, except for the region near the focus which is not essential due to relatively large dimensions of the apical cell. The model described in the present paper assumed the pattern (b), as more appropriate for peri- and anticlinal arrangement of cell walls. However, taking results of the simulation into account, merophytes formed on the basis of (b) are adjusted also to (a).
